# Effects of Resveratrol and Nebivolol on Isolated Vascular and Cardiac Tissues from Young Rats

**DOI:** 10.1155/2014/720386

**Published:** 2014-02-20

**Authors:** Candice Pullen, Fiona R. Coulson, Andrew Fenning

**Affiliations:** School of Medical and Applied Sciences, CQ University, Rockhampton, Australia

## Abstract

The mechanisms by which resveratrol and nebivolol induce vasodilation are not clearly understood. It has been postulated that both agents stimulate the production of nitric oxide; however, this remains to be conclusively established. The major aim of this study was to examine the vasodilatory and antiarrhythmic effects of both resveratrol and nebivolol and to provide further insight into possible mechanisms of action. Cardiac and vascular tissues were isolated from healthy male rodents. Results indicate that resveratrol and nebivolol decrease the action potential duration and induce mild vasorelaxation in aortic and mesenteric segments. Relaxation induced by resveratrol was prevented by the addition of verapamil, N**ω**-nitro-L-arginine-methyl ester, and 4-aminopyridine. This suggests that nebivolol and resveratrol act as putative antiarrhythmic and vasodilatory agents *in vitro* through possible indirect nitric oxide mechanisms.

## 1. Introduction

Currently it is estimated that 3.7 million Australians over the age of 25 have been diagnosed with hypertension [1], making it one of the most common health concerns in Australia. It has been well documented that hypertension has been commonly associated with endothelial dysfunction due, in part, to diminished circulation of nitric oxide [2, 3].

As nitric oxide plays such an important role in maintaining cardiovascular health, much research has been invested in studying various compounds, which may act on or improve the release of nitric oxide [3]. These include nutraceuticals that display novel antioxidant and anti-inflammatory action such as resveratrol and pharmacological agents such as nebivolol. Resveratrol and nebivolol have been demonstrated in both animal and human based studies to increase the bio-availability of nitric oxide [2, 4–6]. These two compounds have also been shown to exhibit beneficial effects on myocardial function by preventing hypertrophy [7, 8] as well as acting to decrease inflammation [9, 10] and oxidative stress [11, 12].

Epidemiological research has cited numerous health benefits related to the regular consumption of red wine. These putative health improvements have been attributed in part to the presence of the antioxidant, resveratrol [2, 13, 14]. Resveratrol has been shown to have not only cardioprotective [8] but also anti-inflammatory [15] and antioxidant properties [2]. Resveratrol's cardioprotective effects, such as improved endothelium function, decreased left ventricular and vascular remodeling, and a decrease in ischemia-induced arryhthmias have been demonstrated in numerous chronic rodent models of diabetes and hypertension [8, 16–18], in patients with coronary artery disease [19] and overweight and/or obese patients [20]. Vasodilatory effects have been observed in isolated rodent mesenteric arteries [21] and in porcine coronary arteries [13]. It has been postulated that in animal models of diabetes and hypertension, resveratrol may be acting to improve nitric oxide release ultimately restoring endothelial function and thus preventing further damage.

Nebivolol is a novel, third generation *β*-blocker, which is administered as a racemic combination of both d- and l-nebivolol. What sets nebivolol apart from other *β*-blockers are its unique haemodynamic properties. Of the two different enantiomers, d-nebivolol has shown to be highly *β*_1_ specific [22], whereas the l-enantiomer has been demonstrated to increase nitric oxide (NO) bioavailability [23]. Research carried out on aortic rings isolated from 10-week-old rats showed that nebivolol was able to induce both endothelial-dependent and NO-dependent relaxation [24]. In human patients, nebivolol may be beneficial in decreasing the risk of developing lethal ventricular arrhythmias, possibly due to modulation of sympathetic hyperactivity [25]. Patients treated with nebivolol had significantly decreased heart rates in comparison to untreated patients [25]. It is therefore possible that nebivolol's antiarrhythmic action may be due to blockade of the *β*_1_-adrenoreceptors. This is further supported by research conducted in numerous animal models and has been associated with various mechanisms including the blockade of the myocardial *β*_1_-adrenoreceptors, reduction of refractory period, and increased release of NO [26].

It remains unclear as to the mechanisms by which resveratrol and nebivolol may be acting to provide the reported protection to the cardiovascular system. Our aim was to investigate the antiarrhythmic and vasodilatory properties of resveratrol and nebivolol and their putative mechanisms of action using healthy cardiac and vascular tissues obtained from rodents.

## 2. Methods

Ethical clearance was granted through Central Queensland University Ethics Committee (AEC 099/11-251). All experimental protocols were carried out using guidelines set out by the committee. Experiments were conducted on male Wistar rats, approximately 9–11 weeks of age. Animals were purchased from the Animal Resources Centre (Perth, Australia) and housed in a 12 hr light/dark cycle under controlled environmental conditions. The animals were permitted a minimum of 1 week to acclimatise to the conditions before euthanasia via an overdose of sodium pentobarbital (375 mg/mL via i.p).

### 2.1. Assessment of Electrophysiological Function

Cardiac electrophysiological function was examined using a protocol similar to previously published studies [11, 27]. The papillary muscle was excised from the left ventricle and a stainless steel hook was inserted through the superior end. The muscle was then placed between two platinum electrodes in a 1.0 mL experimental chamber filled with Tyrode's physiological salt solution (37°C; aerated with 95% oxygen and 5% CO_2_) and fixed into position with a stainless steel pin. The muscle was then slowly stretched to a maximum preload (5–10 mN). The field stimulation was then undertaken using a Grass SD-9 and contractions were induced at 1 Hz, with a pulse width of 0.5 msec and stimulus strength 20% above threshold. After a five-minute equilibration period, the muscle was then impaled by a glass electrode filled with potassium chloride (World Precision Instruments filamented borosilicate glass, outer diameter 1.5 mm, tip resistance of 5–15 mΩ when filled with 3 M potassium chloride). A silver/silver chloride was used as a reference electrode. The electrical activity of a cell was recorded with a Cyto 721 electrometer (World Precision Instruments) connected to an iMAC G5 computer through analogue digital converter (PowerLab 4/25).

After 20 minutes of baseline recordings, tissues were exposed to 30 *μ*M resveratrol (dissolved in Tyrode's solution) followed by exposure to 120 *μ*M resveratrol. Tissues were then rinsed with fresh Tyrode's solution for 10 min and then exposed to 10 *μ*M nebivolol followed by 100 *μ*M nebivolol.

### 2.2. Assessment of Vascular Function

Both thoracic aortic rings and mesenteric arteries were utilised. Aortic and mesenteric segments were freed from any connective or adipose tissue. Eight aortic segments from each animal were suspended in 25 mL organ baths containing Tyrode's solution (37°C; aerated with 95% oxygen and 5% CO_2_). Mesenteric vessels (eight per animal) were suspended in 12 mL myograph chambers containing Tyrode's solution as in the aortic preparation. To ensure consistency, the secondary branch of the mesenteric vessels was utilised for each preparation. All vessels (mesenteric and aortic rings) were allowed to equilibrate for a minimum of 30 minutes, after which the mesenteric vessels were normalised using the standard DMT normalisation process [28].

Half of the tissues from each animal were precontracted with Tyrode's solution containing 10 mM potassium chloride. Once the contractile response had plateaued, relaxation response curves were generated to either resveratrol (1e^−8^–3e^−4^ M) or nebivolol (1e^−8^–3e^−5^ M). It is important to note that if a relaxation response was induced by one of the above-mentioned compounds, the response was allowed to plateau before the next dose of the compound was added. If no response was induced after approximately 3 minutes, the next dose was added to the baths. Following this, the remaining tissues were precontracted with noradrenaline (3e^−7^ M) and relaxation response curves were generated to either resveratrol (1e^−8^–3e^−4^ M) or acetylcholine (1e^−8^–3e^−4^ M). Once completed, all tissues were rinsed repeatedly with fresh Tyrode's solution and were given 30 minutes to equilibrate back to baseline. The tissues were then precontracted a second time using Tyrode's solution containing 5 mM potassium chloride. The relaxation response curves to resveratrol and nebivolol were repeated but in the presence of one of the following antagonists: 3e^−7^ M verapamil, 1e^−5^ M N*ω*-nitro-L-arginine-methyl ester(L-NAME), or 1e^−3^ M 4-aminopyridine (resveratrol only).

### 2.3. Statistical Analysis

Data are expressed as mean ± standard error mean (SEM). Statistical analysis was performed using one-way analysis of variance (ANOVA) with a Bonferroni posttest and Student's *t*-test. Results were considered significant when *P* < 0.05. Analysis was carried out using Graphpad Prism v4 (GraphPad Software, CA, USA).

### 2.4. Drugs

All drugs used were purchased from Sigma Aldrich (St. Louis, MO, USA). Resveratrol and nebivolol were dissolved in dimethyl sulfoxide (DMSO) to a stock concentration of 10^−1^ M. The final concentration of DMSO in the organ baths was not sufficient to induce any physiological response in the tissues tested. Noradrenaline, acetylcholine, L-NAME, 4-aminopyridine, and verapamil were all dissolved in distilled water to a stock solution of 1 M.

## 3. Results

### 3.1. Electrophysiological Function

**Figure 1:**
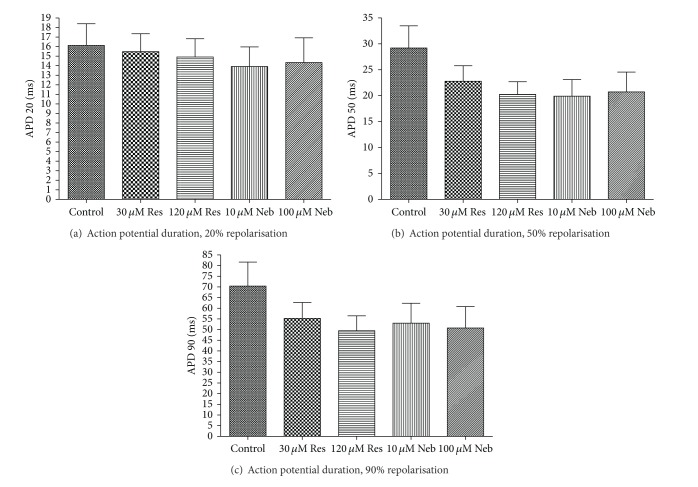
Effects of various doses of resveratrol and nebivolol on the action potential duration in normal rat papillary muscles. Res: resveratrol; Neb: Nebivolol. (a) Action potential duration at 20% of repolarisation. (b) Action potential duration at 50% of repolarisation. (c) Action potential duration at 90% of repolarisation. Data presented as mean ± SEM; *n* = 5–8.

Resveratrol did not significantly alter the resting membrane potential, action potential amplitude, or the force of contraction recorded from the left ventricular papillary muscles (Table 1). Resveratrol did display a trend to dose dependently decrease the action potential duration at all three-time points analysed; however, this trend was more pronounced at 50% and 90% of repolarisation (Figure 1) but did not reach statistical significance.

Similar results were seen after the addition of nebivolol. There were no significant changes in the resting membrane potential, action potential amplitude, or force of contraction after exposure to nebivolol (Table 1). As with resveratrol, nebivolol displayed a nonsignificant trend to decrease action potential duration; however, these effects were not dose dependent (Figure 1).

### 3.2. Vascular Function

#### 3.2.1. Thoracic Aorta

**Figure 2:**
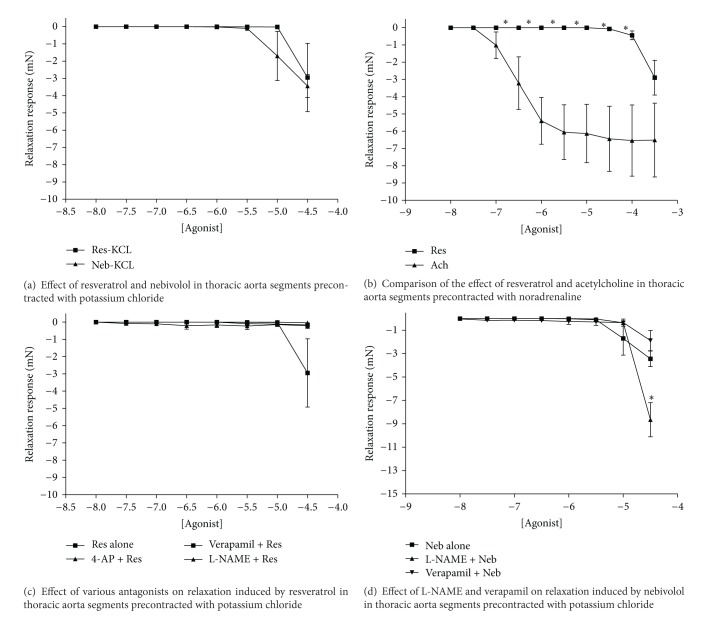
Vasorelaxation induced by the addition of either resveratrol or nebivolol in isolated thoracic aortic segments. KCL: tydrodes solution containing potassium chloride; Res: resveratrol; Neb: nebivolol; Ach: acetylcholine; 4-AP: 4-aminopyridine; L-NAME: N*ω*-nitro-L-arginine-methyl ester. *P < 0.05; data presented as mean ± SEM; *n* = 9 for all groups.

When precontracted with both potassium chloride and noradrenaline, resveratrol only induced relaxation at high doses (Figures 2(a) and 2(b)). It was not as effective as acetylcholine in relaxing the precontracted aortic tissues (Figure 2(b)). The addition of L-NAME, 4-aminopyridine, and verapamil abolished the response to resveratrol (Figure 2(c)). Nebivolol also acted as a moderate vasodilator in aortic tissue (Figure 2(a)). Neither L-NAME nor verapamil significantly altered the relaxation response due to nebivolol (Figure 2(d)).

#### 3.2.2. Mesenteric Vessels

**Figure 3:**
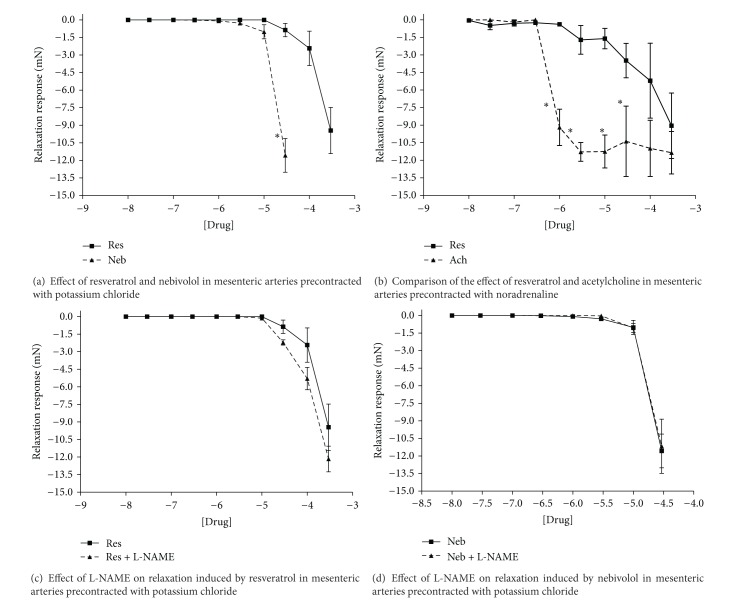
Relaxation responses induced by the addition of either resveratrol or nebivolol in isolated segments of mesenteric arteries. Res: resveratrol; Neb: nebivolol; Ach: acetylcholine; L-NAME: N*ω*-nitro-L-arginine-methyl-ester. *P < 0.05, data presented as mean ± SEM; *n* = 3–6.

Resveratrol was more effective at inducing a relaxation response, in the mesenteric vessels, when precontracted with noradrenaline than with potassium chloride (Figures 3(a) and 3(b)). However, as with the aorta, resveratrol was not as effective as acetylcholine in producing a relaxation response (Figure 3(b)). The addition of L-NAME did not significantly inhibit the resveratrol-induced relaxation (Figure 3(c)). Nebivolol was only able to induce relaxation in potassium chloride precontracted tissues at relatively high doses (Figure 3(a)). The addition of the nitric oxide synthase inhibitor, L-NAME, did not affect the relaxation induced by nebivolol (Figure 3(d)).

## 4. Discussion

The major aim of this study was to investigate the possible antiarrhythmic and vasodilatory role of both resveratrol and nebivolol on isolated tissues. The electrophysiological studies demonstrated that both nebivolol and resveratrol have the potential to reduce action potential duration. This trend, though found not to be significant, was seen at both 50 and 90% of repolarisation. There was no significant effect on the force of the contraction, resting membrane potential, or the action potential amplitude after treatment with either compound.

Resveratrol and nebivolol acted as moderate vasodilators on both large conduit arteries (aorta) and small resistance vessels (mesenteric arteries). These effects were only noted at relatively high concentrations and appear to be more subtle in healthy tissues compared to those reported in diseased models. The addition of various antagonists inhibited resveratrol's effect in the aortic segments, but not in the mesenteric vessels. The relaxant effect of nebivolol was not hampered by the addition of either L-NAME or verapamil in both types of blood vessels studied.

### 4.1. Electrophysiological Function

Resveratrol's effect on cardiomyocytes remains unclear. Some studies report that exposure to resveratrol decreases the action potential duration (APD) in isolated cardiomyocytes [29, 30], whereas others report that resveratrol prolongs the APD [16]. Although the literature indicates conflicting results, the consensus is that resveratrol mediates its electrophysiological effects via alterations in ionic calcium and potassium channels [5, 16, 29, 30].

In our study, both resveratrol and nebivolol decreased the duration of the potentials recorded from the isolated papillary muscles at both 50% and 90% of repolarisation. In the case of resveratrol, these effects appear to be dose dependent. Although the results were not deemed to be significant after statistical analysis, the trends are noteworthy. There was no alteration in either the resting membrane potential, action potential amplitude, or the force of contraction after the muscles were exposed to either resveratrol or nebivolol indicating that there were minimal changes in basal cell function.

The decreases in action potential duration after exposure to resveratrol, seen in the current study, are similar to those observed in the guinea pig which concluded that resveratrol may inhibit calcium influx in myocytes [30]. As the duration of repolarisation is heavily dependent on potassium efflux and calcium influx, it would be expected that inhibition of calcium influx would result in a faster repolarisation of the cardiomyocytes. These findings are further supported by patch-clamp studies carried out in ventricular myocytes isolated from male Sprague-Dawley rats [31]. In the rodent myocytes it is found that resveratrol inhibited the activation of the L-type calcium channel, which may account, at least in part, for resveratrol's acute antiarrhythmic actions [31] and also contribute to its vascular function.

Similar alterations in the action potential were seen after the papillary muscles were exposed to nebivolol. At this stage, it is unclear as to the exact mechanism that may be at play behind the decrease in action potential duration after nebivolol exposure. A related study examining the negative inotropic effects of the *β*_1_-selective adrenoceptor blocker esmolol found similar results [32]. The authors proposed that the mechanism behind esmolol's effects was through the inhibition of the calcium current [32]. The reduction in action potential duration by esmolol was also accompanied by a reduction in the force of the contraction [32]. This can be explained through the inhibition of the calcium current resulting in less calcium available intracellularly to initiate a contractile response. These findings are consistent with clinical studies investigating long QT syndrome. Long QT syndrome is considered a clinical marker for ventricular arrhythmia and end-organ damage in hypertensive patients. The reduction of QT dispersion induced by nebivolol treatment has been reported to be independent of a decrease in blood pressure [25].

### 4.2. Vascular Function

Our study suggests that the resveratrol has the ability to induce mild relaxation in vascular tissue; however, the effects were more pronounced in the small resistance vessels than in the large aortic vessels precontracted with noradrenaline. In aortic tissues precontracted with potassium chloride, 4-aminopyridine, L-NAME, and verapamil completely abolished the relaxation induced by resveratrol, suggesting that the vasodilation induced by resveratrol in large conduit arteries, such as the aorta, may be both endothelium dependent and independent. However, L-NAME appeared to potentiate the vasorelaxation response induced in small resistance vessels.

There is much debate in the current literature regarding the exact mechanism by which resveratrol induces vasorelaxation. In retinal arterioles, resveratrol induced relaxation in a dose-dependent manner [14]. The authors found that the addition of L-NAME partially prevented the resveratrol-mediated relaxation [14], suggesting that the activation of endothelial nitric oxide synthase may be partially responsible for the vasorelaxation. However, other published studies have speculated that potassium channels may play a role [21, 33]. In studies conducted in isolated rat aortic rings, the presence of 4-aminopyridine and magatoxin (a Kv1 channel inhibitor) completely abolished the resveratrol-induced vasorelaxation [33]. Similar results have been reported in mesenteric arteries collected from healthy rats [21]. The possibility exists that the discrepancies reported regarding resveratrol's mechanism of action may be due to the fact that responses may vary between healthy and diseased tissue, and in acute versus chronic administration.

Data gained in our study demonstrated that nebivolol induced mild relaxation in both the aortic and mesenteric vessels that were precontracted with potassium chloride. The addition of either L-NAME or verapamil did not appear to hinder the relaxation produced by nebivolol. These results suggest that the response exhibited was not due to the release of nitric oxide or mediated through calcium channels. Adding nebivolol directly to the vascular tissue effectively bypassed metabolism; it is possible that it is the metabolites of nebivolol that are responsible for the reported effect on nitric oxide. This conclusion is supported by previously published studies [22]. A 50/50 mixture of both d- and l-nebivolol, once metabolised, was demonstrated to induce the release of nitric oxide in mouse aortas. These effects were not seen with nonmetabolised nebivolol or nebivolol bound to plasma [22]. The same study also found that even if nebivolol had been metabolised, it was still unable to induce an increase in NO if there was an insufficient amount of either intracellular or extracellular calcium.

The mechanisms by which nebivolol induces vasodilation are more established than resveratrol. Current literature places a heavy emphasis on the beneficial effect that nebivolol has not only on cardiovascular function but also on providing protection to the endothelium [4]. Patients with coronary artery disease treated with nebivolol for four weeks displayed an improvement in endothelium-dependent vasodilation in the brachial artery [34]. The authors suggest that these improvements were due to an increase in nitric oxide production and/or bioactivity [34].

One limitation of this study was the use of verapamil as a calcium channel antagonist in the vascular tissue. A more appropriate choice may have been a compound such as nifedipine (vascular selective) or diltiazem (mixed cardiac and vascular effects). Verapamil has been shown to antagonise calcium channels in both cardiac and vascular tissues [35]. As this study examines various responses in both cardiac and vascular tissues, it was necessary to select a compound that would act on calcium channels in both experimental protocols. Verapamil was selected in order to explore any common effects.

## 5. Conclusion

Our results agree with the previously published data suggesting that both resveratrol and nebivolol display antiarrhythmic properties as demonstrated in their ability to decrease the duration of the cardiac action potential. Both compounds only elicit minor relaxation responses in both conduit and small resistance vessels at high concentrations. Drinking red wine alone would not provide a high enough concentration of resveratrol to benefit from its vasodilatory effects as red wine only contains 1.5–3.0 mg/L of resveratrol [36]; however, it can be achieved through supplementation.

In the aorta, resveratrol-induced relaxation was mediated mainly through independent mechanisms. The vasorelaxation response induced by nebivolol was not nitric oxide mediated. Both resveratrol and nebivolol displayed relatively mild effects when added acutely to healthy tissues. This effect may be more significant when examined in a chronic and/or diseased model as both compounds have been demonstrated to improve oxidative stress, decrease inflammation, and provide protection to the delicate endothelial lining of blood vessels.

## Figures and Tables

**Table 1:** Effect of resveratrol and nebivolol on resting membrane potential (RMP), action potential amplitude (APA) and force of contraction (FOC) in isolated papillary muscle cells.

	Control	30 *μ*M Res	120 *μ*M Res	10 *μ*M Neb	100 *μ*M Neb
RMP	−62.2 ± 1.9 n = 8	−63.5 ± 2.5 n = 5	−65.5 ± 1.7 n = 4	−66.3 ± 2.4 n = 4	−65.5 ± 1.1 n = 4
APA	69.7 ± 6.1 n = 8	65.7 ± 4.3 n = 5	66.9 ± 4.3 n = 4	65.4 ± 8.7 n = 4	68.4 ± 9.3 n = 4
FOC	1.2 ± 0.4 *n* = 8	1.2 ± 0.3 n = 5	1.5 ± 0.6 n = 4	1.9 ± 0.5 n = 4	2.0 ± 0.6 n = 4
